# Identification of Human NK17/NK1 Cells

**DOI:** 10.1371/journal.pone.0026780

**Published:** 2011-10-21

**Authors:** Abhilash D. Pandya, Zaidoon Al-Jaderi, Rune A. Høglund, Trygve Holmøy, Hanne F. Harbo, Johannes Norgauer, Azzam A. Maghazachi

**Affiliations:** 1 Department of Physiology, Faculty of Medicine, Institute of Basic Medical Sciences, University of Oslo, Oslo, Norway; 2 Institute of Immunology, Oslo University Hospital and University of Oslo, Oslo, Norway; 3 Department of Neurology, Oslo University Hospital and University of Oslo, Oslo, Norway; 4 Department of Dermatology, Jena University Hospital, Jena, Germany; Innsbruck Medical University, Austria

## Abstract

**Background:**

Natural killer (NK) cells have both cytolytic and immunoregulatory functions. We recently described that these cells release the inflammatory cytokines IL-17 and IFN-γ. However, the precise identity of the NK cell subset(s) that secrete these cytokines is not known.

**Methodology/Principal Findings:**

To isolate the cells secreting IL-17 and IFN-γ, we took advantage of the findings that Th17/Th1 cells express chemokine receptors. Therefore, CD56^+^NK cells were stained with antibodies against various chemokine receptors and intracellularly with antibodies toward IL-17 and IFN-γ. Consequently, we identified previously unrecognized subset of NK cells generated from normal human peripheral blood after activation with IL-2 but not PMA plus ionomycin. The cells are characterized by the expression of CD56^+^ and CCR4^+^, produce IL-17 and IFN-γ and are consequently named NK17/NK1 cells. They also express CD161, NKp30, NKp44, NKp46, NKG2D, CD158, CCL22, IL-2Rβ and the common γ chain but not CD127 or IL-23R. Further, they possess T-bet and RORγt transcription factors. Antibodies to IL-1β, IL-6, IL-21, or TGF-β1 do not inhibit IL-2-induced generation of NK17/NK1 cells, suggesting that IL-2 has the capacity to polarize these cells. Notably, NK17/NK1 cells are abundant in the cerebrospinal fluid (CSF) of patients with multiple sclerosis (MS) without activation, and are generated from the peripheral blood of these patients after activation with IL-2.

**Conclusions/Significance:**

NK17/NK1 cells identified here have not been previously described in healthy or MS patients.

## Introduction

Natural killer (NK) cells represent the first line of defence against infections and tumor metastases [1]. These cells possess immunoregulatory activities by secreting multiple cytokines and chemokines, and interact with dendritic cells to shape the innate and adaptive immune responses. Traditionally, human NK cells are classified into two major subsets; regulatory cells expressing CD56 but not CD16 known as CD56^+/high^CD16^−^, and cytolytic cells expressing CD16 and low or no CD56 known as CD56^−/low^CD16^+^[reviewed in 2]. In addition, NK cells have been classified into NK1 and NK2 subsets based on cytokine release [3], and divided into different subsets based on their expression of chemokine receptors [4].

A unique subset of NK cells lining human peyers patches or tonsils that express NKp44 and CCR6 has been also described. The cells have no cytotoxic granules, do not secrete IFN-γ and IL-17, but secrete IL-22, and were consequently designated as “NK22” cells [5]. Similar cells were reported by Cupedo et al. who demonstrated that cells with lymphoid tissue inducers (LTi) phenotype, i.e. CD127^+^, lymphotoxin^+^ and the nuclear factor retinoic acid-related orphan receptor (RORC^+^), can differentiate into cells secreting IL-22 and expressing the CD56^+^CD127^+^RORC^+^ phenotype [6]. Also, NKp46^+^ NKG2D^+^ NK1.1^int^ RORγt^high^ NK cells in intestinal lamina propria were found to secrete the Th17 cytokine IL-22 [7]. In tonsil tissues, NK cells in stage III development expressing CD34^+^ CD117^+^ 2B4^+^phenotype, as well as secreting IL-22 and IL-26 but not IL-17, have also been described [8]. Collectively, these observations identified NK cells found at mucosal tissues that secrete IL-22 and express among many markers RORγt and CCR6. These findings also suggest that NK cells may be involved in autoimmune diseases by releasing inflammatory cytokines such as IL-17 and IL-22.

The role of NK cells in autoimmune diseases has not been delineated with precision. It was suggested that these cells play important roles in these diseases and they could be targets for therapy [9]. However, the role of NK cells in multiple sclerosis (MS) is controversial as there are two schools, one indicates that NK cells ameliorate the disease, whereas the other suggests that they exacerbate it [reviewed in 10]. It was reported that IL-2-activated NK cells release IL-17 and IFN-γ [11], [12], but the identity of the cells that secrete these cytokines and their relation to the recently described NK cells in the gut mucosa or tonsils are not known. In fact, very little is known about the different subsets of NK cells and the function of these subsets. The purpose of this report is to isolate and characterize NK cells that secrete IL-17 and IFN-γ from normal individuals and from patients with MS.

## Materials and Methods

### Antibodies

PE-conjugated mouse anti-NKp30 (CD337), PE-conjugated mouse anti-NKp44 (CD336), PE-conjugated mouse anti-NKp46 (CD335), PE-conjugated mouse anti-NKG2D (CD314), PE-conjugated mouse anti-CD161, FITC-conjugated anti-CD3, and FITC-conjugated anti-CD19 and PE-conjugated IgG1 isotype control were purchased from Becton-Dickinson (San Diego, CA). FITC-conjugated mouse anti-IL-17A, APC-conjugated anti-IL17, PE-conjugated rat anti-RORγt, PE-conjugated rat anti-T-bet, mouse and rat IgG isotype controls were purchased from eBioscience (San Diego, CA, USA). Mouse anti-CCR4, mouse anti-CCR6, mouse anti-CCR7, mouse anti-CXCR3, FITC-conjugated mouse anti-CCR4, FITC-conjugated mouse anti-CCR6, FITC-conjugated mouse anti-CCR7, FITC-conjugated mouse anti-CCR9, FITC-conjugated mouse anti-CXCR1, FITC-conjugated mouse anti-CXCR3, FITC-conjugated anti-mouse CXCR4, PE-conjugated mouse anti-CD158, PE-conjugated mouse anti-CD127 (IL-7Rα), PE-conjugated mouse anti-CD132 (common γ chain), PE-conjugated mouse anti-IL-2Rβ, PE-conjugated mouse anti-IL-17, PE-conjugated mouse anti-IFN-γ, PE-conjugated mouse anti-IL-23R, PE-conjugated mouse anti-CCL22 (MDC), mouse anti-human mouse IL-1β, mouse anti-human IL-6, goat anti-human anti-TGF-β1, mouse IgG_1_, mouse IgG_2B,_ mouse IgG_2A_, rat and goat IgG were all purchased from R&D Systems (R&D Systems Europe Ltd., Abingdon, UK). FITC-conjugated anti-CD14 and FITC-conjugated anti-CD56 were obtained from Immunotools (Friesoythe, Germany).

### NK cell isolation

Buffy coats from healthy volunteers were obtained from the blood bank (Ullevål Hospital, Oslo, Norway). NK cell isolation was performed using RosetteSep human NK cell enrichment cocktail (Stem cell technologies SARL, Grenoble, France). Approximately 50 mL buffy coat was diluted 1∶1 with RPMI medium and incubated with 25 µL of the cocktail provided with the kit for 20 min at room temperature. Afterwards, the mixture was centrifuged at 1800 rpm for 25 min, using Histopaque (Sigma-Aldrich, Oslo, Norway), and NK cell layer collected. The cells were further sorted into CD56^+^ and CD56^−^ cells by magnetic separation, using EasySep human CD56 positive selection kit (Stem cell technologies SARL). After separation both CD56^+^ and CD56^−^ cells were collected. To activate the cells, NK cells isolated by RosetteSep human NK cell enrichment cocktail (not yet separated with CD56 cocktail), were incubated at 1×10^6^/mL with 200 U/mL IL-2. IL-2 (200 U/mL) was added to the cultures after 2, 4 and 6 days. The cells were collected after 7 days and then separated into CD56^+^ and CD56^−^. Activation with Phorbol 12-myristate 13-acetate (PMA) and ionomycin (both from Sigma-Aldrich, Oslo, Norway) was done by incubating isolated CD56^+^cells with 100 ng/mL of PMA and Ionomycin each for 24 h. Cells were washed and stained with surface anti-CCR4, and intracellularly with anti-IL-17 and anti-IFN-γ. CCR4 gated cells were then examined for the expression of IL-17 and IFN-γ.

To isolate CCR4^+^ or CCR4^−^, sorted CD56^+^ NK cells (1×10^6^/mL) were mixed with 1 µg/ mL of anti-CCR4 in 12 mL tubes, and the mixtures were incubated for 45 min at 4°C. The cells were washed twice with PBS plus 1% BSA, and incubated with goat anti-mouse DYNAL® magnetic beads (Invitrogene, Oslo, Norway) for 60 min at 4°C. Cells that attached to the beads and those that did not attach were isolated and tested for purity by flow cytometry.

### Flow cytometric analysis

For single color surface analysis, 3×10^5^ cells/well were labeled in the dark at 4°C for 45 min with 0.1 µg/well FITC-conjugated anti-CD3, 0.15 µg/well FITC-conjugated anti-CD14 and 0.1 µg/well FITC-conjugated anti-CD19, 0.06 µg/well PE-conjugated anti-CD127, 0.06 µg/well PE-conjugated anti-CD158, 2.5 µL PE-conjugated anti-CD161, 0.12 µg/well PE-conjugated anti-IL-23R, 0.5 µg/well anti-NKp30, 0.5 µg/well PE-conjugated anti-NKp44, 0.5 µg/well PE-conjugated anti-NKp46, 0.5 µg/well PE-conjugated anti-NKG2D, 0.06 µg/well PE-conjugated anti-IL-2Rβ or isotype control antibodies. To stain intracellular molecules, cells were fixed with 4% paraformaldehyde for 15 min at 4°C, washed twice with SAP buffer and then labeled with 0.5 µg/well PE-conjugated anti-RORγt, 0.5 µg/well PE-conjugated anti-T-bet, 0.12 µg/well PE-conjugated anti-CCL22, 0.06 µg/well PE-conjugated anti-IL-2Rγ or isotype control antibodies. Gating was done according to the isotype controls. The cells were washed and then examined in the flow cytometry ((FACSCalibur, Becton Dickinson Biosciences, San Jose, CA).

To satin with the chemokine receptor and one intracellular cytokine, cells were incubated with 10 µg/mL Brefeldin A for 4 hours. They were labeled at 3×10^5^ cells/200 µL/well with 0.06 µg/well FITC labeled anti-CCR4, 0.06 µg/well FITC-conjugated anti-CCR6, 0.06 µg/well FITC-conjugated anti-CCR7, 0.25 µg/well FITC-conjugated anti-CCR9, 0.12 µg/well FITC-conjugated anti-CXCR1, 0.12 µg/well FITC-conjugated anti-CXCR3, 0.12 µg/well FITC-conjugated anti-CXCR4, or isotype control antibodies for 45 min at 4°C in the dark. After incubation, the cells were fixed with 4% paraformaldehyde for 15 min at 4°C and then washed twice with SAP buffer before staining with intracellular markers as follows: 3×10^5^ cells/well were incubated with 0.06 µg/well PE-conjugated anti-IL-17, 0.06 µg/well anti-PE-conjugated IFN-γ, 0.06 µg/well PE-conjugated anti-CCL3, 0.06 µg/well PE-conjugated anti-CCL4 or isotype controls antibodies, in the dark at 4°C for 45 min. Cells were washed with flow cytometric medium, resuspended with the same medium and transferred from plates into 5 ml tubes to perform flow cytometric analysis. Compensation was done according to the isotype controls. Analysis was done by FlowJo (Flow cytometry analysis software, Ashland, OR, USA).

For three color analysis, 1×10^6^ cells/well were labeled with 0.125 µg/well FITC-conjugated anti-CCR4, 0.125 µg/well FITC-conjugated anti-CCR6, 0.125 µg/well FITC-conjugated anti-CCR7, 0.3 µg/well FITC-conjugated anti-CCR9, 0.2 µg/well FITC-conjugated anti-CXCR4 or control FITC-conjugated IgG antibodies at 4°C for 45 min in the dark. These cells were fixed with 4% paraformaldehyde for 15 min at 4°C and then washed twice with SAP buffer before staining them with intracellular markers as such: 0.04 µg/well APC-conjugated anti-IL-17, 0.1 µg/well PE-conjugated anti-IFNγ or isotype controls antibodies were added in the dark at room temperature for 45 min. The cells were washed with flow cytometric buffer and resuspended in the same buffer. FITC-conjugated cells (more than 99% pure) were gated and examined for the production of IL-17 and IFNγ.

### Treatment with the antibodies

Enriched NK cells were incubated with IL-2 as described above, in the absence or the presence of these neutralizing antibodies: 1 µg/mL anti-IL-1β, anti-IL-6, anti-IL-21, anti-TGF-β1 or isotype control antibodies for 6–7 days. The cells were collected, washed and CD56^+^ cells isolated. They were labeled extracellularly with anti-CCR4 and intracellularly with anti-IL-17 and anti-IFN-γ, and then examined in the flow cytometry.

### Multiple Sclerosis patients

The local ethical committee at Ullevål Hospital and Oslo University Hospital approved the study, and patients were informed and signed consent forms according to the approved protocol. All patients fulfilled McDonald's diagnostic criteria. Five patients with relapsing remitting (RR) MS diagnosis donated blood samples in a clinical stable phase of the disease and before receiving any treatment. Three other patients donated CSF (Table 1). Peripheral blood cells from these patients were incubated with IL-2 similar to normal blood. Cells from the CSF were sorted into non-activated CD56^+^ and CD56^−^, and were labeled with surface anti-CCR4 and intracellularly with IL-17 and IFN-γ, as described above. Cells isolated from the CSF of a third MS patient with secondary progressive MS (Table 1) were labeled with FITC-conjugated anti-CCR4, fixed, permeabilized and stained with PE-conjugated anti-IFN-γ and APC-conjugated anti-IL-17. They were examined by flow cytometry as described above.

**Table 1:** MS patients examined in this study.

Year born	Sample	Sex	Disease course
1958	PB	Female	RRMS
1972	PB	Male	RRMS
1963	PB	Female	RRMS
1972	PB	Female	RRMS
1973	PB	Male	RRMS (early secondary progressive MS)
1964	CSF	Female	RRMS
1986	CSF	Female	RRMS
1961	CSF	Male	Secondary progressive MS

PB = peripheral blood. CSF = cerebrospinal fluid. RRMS = relapsing-remitting MS.

### Detection of IL-17 and IFN-γ levels by ELISA assay

Concentrations of IL-17 and IFN-γ were determined with the human Quantikine ELISA kits (R&D Systems Europe Ltd) as described by the manufacturers' user manual. Supernatants from IL-2-activated CD56^+^CCR4^+^ NK cells (5×10^5^ or 1×10^6^ cells/mL), were collected and the levels of IFNγ and IL-17 were determined at 450 nm with Power wave XS plate reader (Biotec instruments, VT, USA).

### Statistical analysis

Significant values were generated by the Student *t*-test utilizing GraphPad Prism 3 software (GraphPad Software, Inc., La Jolla CA, USA). A P value<0.05 was considered to be statistically significant.

## Results

### IL-2-activated CD56^+^CCR4^+^ NK cells produce and secrete IL-17 and IFN-γ

To investigate the presence of NK cells secreting IL-17 and IFN-γ in human peripheral blood, we used an approach based on the finding that chemokine receptor CCR6 is expressed on cells secreting IL-17 or IL-17 plus IFN-γ [13], [14]. First, we isolated non-activated NK cells from normal human blood and sorted them into CD56^+^ and CD56^−^ using antibody-coated beads. The surface of highly purified CD56^+^ and CD56^−^NK cells were labeled with FITC-conjugated anti-CCR6. Because NK cells also express CCR4 [15], and because this molecule is present on Th17 cells in addition to CCR6 [13], [14], we stained non-activated CD56^−^ and CD56^+^ NK cells with anti-CCR4. NK cells express most other chemokine receptors that are involved in their chemotaxis, migration and cytotoxicity. For example, they express CXCR4 important for their chemotaxis and retention in the bone marrow [16], [17], CCR6 is reported to increase their migration [18], [19], CCR7 is important for their migration and lodging into the lymph nodes [20], [21], and CCR9 involved in migration of cells into the small intestine is expressed on a subset of NK cells [4]. Consequently, we labeled these cells with antibodies to CCR6, CCR7, CCR9 and CXCR4 receptors. All these subsets of NK cells were stained intracellularly with PE-conjugated antibody to IL-17 and IFN-γ. The results demonstrate that less than 2% of non-activated CD56^+^ NK cells, or CD56^−^K cells that were labeled with antibodies toward CCR4, CCR6, CCR7, CCR9 or CXCR4 produced IL-17 or IFN-γ (data not shown).

**Figure 1:**
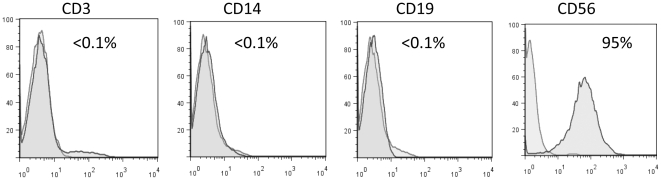
Phenotypes of CD56^+^ cells. IL-2-activated CD56^+^ cells were isolated by EasySep human CD56 positive selection kit. They were examined for the expression of CD3, CD14, CD19 and CD56. Background control using isotype antibodies are also shown. Numbers indicate the percentage of positive cells. One of 5 experiments performed.

**Figure 2:**
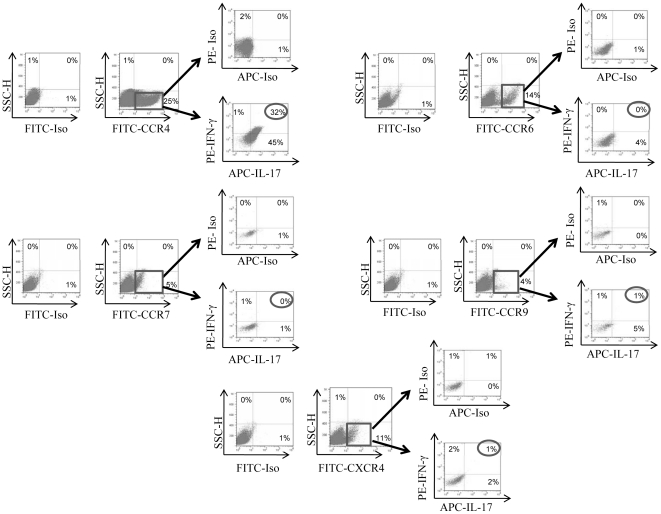
Only CD56^+^CCR4^+^ produce IL-17 and IFN-γ. CD56^+^ NK cells were labeled with FITC-conjugated isotype control antibodies or FITC-conjugated anti-CCR4, anti-CCR6, anti-CCR7, anti-CCR9 or anti-CXCR4 antibodies. They were also labeled intracellularly with PE-conjugated and APC-conjugated isotype control antibodies, or PE-conjugated anti- IFN-γ and APC-conjugated anti-IL-17 antibodies. FITC-conjugated cells were gated (more than 99% pure for the expression of a particular chemokine receptor) and examined for the production of IL-17 and IFN-γ. Labeling with isotype control antibodies is also shown. Numbers show the percentage of positive cells. This is a representative experiment of five different donors.

**Figure 3:**
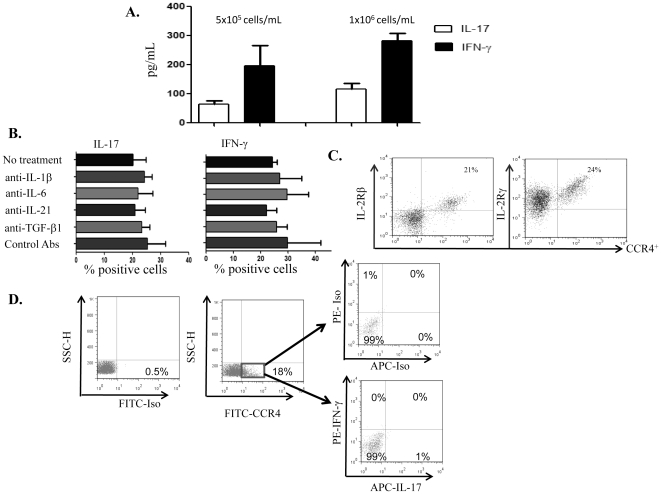
IL-2 induces the polarization of NK17/NK1 cells. A) Supernatants were collected from different cell numbers (5×10^5^ and 1×10^6^/mL) of CD56^+^CCR4^+^ cells, and examined for the levels of IL-17 and IFN-γ by ELISA. Mean±SEM of three experiments. B) Enriched CD56^+^ NK cells were incubated with IL-2 only (no treatment), or with IL-2 plus neutralizing antibodies to IL-1β, IL-6, IL-21, TGF-β1, or a combination of isotype control goat and mouse antibodies for 6 days. The cells were collected, and labeled with FITC-conjugated anti-CCR4 and intracellularly with PE-conjugated antibodies to IL-17 and IFN-γ. Mean±SEM of positive cells collected from the blood of three donors. C) CD56^+^ NK cells were labeled with isotype control (not shown) or FITC-conjugated anti-CCR4 and PE-conjugated anti-IL-2Rβ antibodies or intracellularly with PE-conjugated anti-common γ chain (IL-2Rγ). Numbers show the percentages of positive cells. D) CD56^+^ NK cells were incubated overnight with 100 ng/mL PMA plus ionomycin. FITC-conjugated CCR4 cells were gated and examined for the production of IL-17 and IFN-γ. Labeling with isotype control antibodies is also shown. Numbers show the percentages of positive cells.

Since NK cells have been shown to secrete these cytokines upon IL-2 activation [11], [12], we activated purified NK cells *in vitro* with IL-2 for 7 days and then sorted them into CD56^+^ and CD56^−^ subsets. First, we ascertained that the CD56^+^ NK cells are pure since they were stained with anti-CD56 and not with anti-CD3 “T cells”, anti-CD14 “monocytes”, or anti-CD19 “B cells” (Figure 1). Cells of both CD56^+^ and CD56^−^ subsets were labeled with surface anti-CCR4, anti-CCR6, anti-CCR7, anti-CCR9, or anti-CXCR4 and intracellularly with antibodies toward IL-17 and IFN-γ. There was an upregulation of IL-17 and IFN-γ in CCR4^+^CD56^+^ and not CD56^−^ cells (data not shown). Based on these preliminary findings, we examined IL-2 activated CD56^+^ cells labeled with antibody towards CCR4, as well as with anti-CCR6, anti-CCR7, anti-CCR9 or anti-CXCR4. As shown in Figure 2, about 25%, 14%, 5%, 4% and 11% of IL-2 activated CD56^+^ NK cells were stained with FITC-conjugated anti-CCR4, anti-CCR6, anti-CCR7, anti-CCR9 and anti-CXCR4, respectively, whereas isotype control antibodies did not label these cells. FITC-labeled cells were gated (more than 99% pure), and stained intracellularly with PE-conjugated anti-IFN-γ and APC-conjugated anti-IL-17 or with isotype controls PE-conjugated and APC-conjugated antibodies. The results demonstrate that only CCR4^+^ and not CCR6^+^, CCR7^+^, CCR9^+^ or CXCR4^+^ NK cells were labeled intracellularly with antibodies to both cytokines (Figure 2). These results indicate that cells contained within the CD56^+^CCR4^+^ NK cell subset are primary targets for polarization into cells producing IL-17 and IFN-γ, designated here as NK17/NK1 synonymous with T cell terminology [14]. In addition to flow cytometric analysis, we measured the levels of these cytokine in the supernatants of NK17/NK1 cells. Results in Figure 3A show that high levels of both cytokines are released from 5×10^5^/mL or 1×10^6^/mL CD56^+^CCR4^+^ cells.

Incubating purified NK cells with IL-2 in the absence or presence of neutralizing antibodies to IL-1β, IL-6, IL-21, or TGF-β1 did not affect the percentages of CD56^+^CCR4^+^ cells secreting both IL-17 and IFN-γ (Figure 3B), implicating that among these targeted cytokines only IL-2 has the capacity to polarize these cells. To demonstrate that NK17/NK1 cells express IL-2R, sorted CD56^+^cells were labeled with FITC-conjugated anti-CCR4 and PE-conjugated anti-IL-2Rβ or intracellularly with PE-conjugated anti-IL-2Rγ (common γ chain). Data shown in Figure 3C indicate that CD56^+^CCR4^+^ cells expressed both IL-2Rβ and IL-2Rγ. Further analysis showed that incubating CD56^+^ NK cells with stimuli other than IL-2 such as PMA plus ionomycin overnight did not generate cells that produce IL-17 plus IFN-γ, albeit the presence of 18% CCR4^+^ cells within the CD56^+^ cell population (Figure 3D).

### NK17/NK1 cells do not express IL-23R but express RORγt, T-bet and NK cell maturation markers

**Figure 4:**
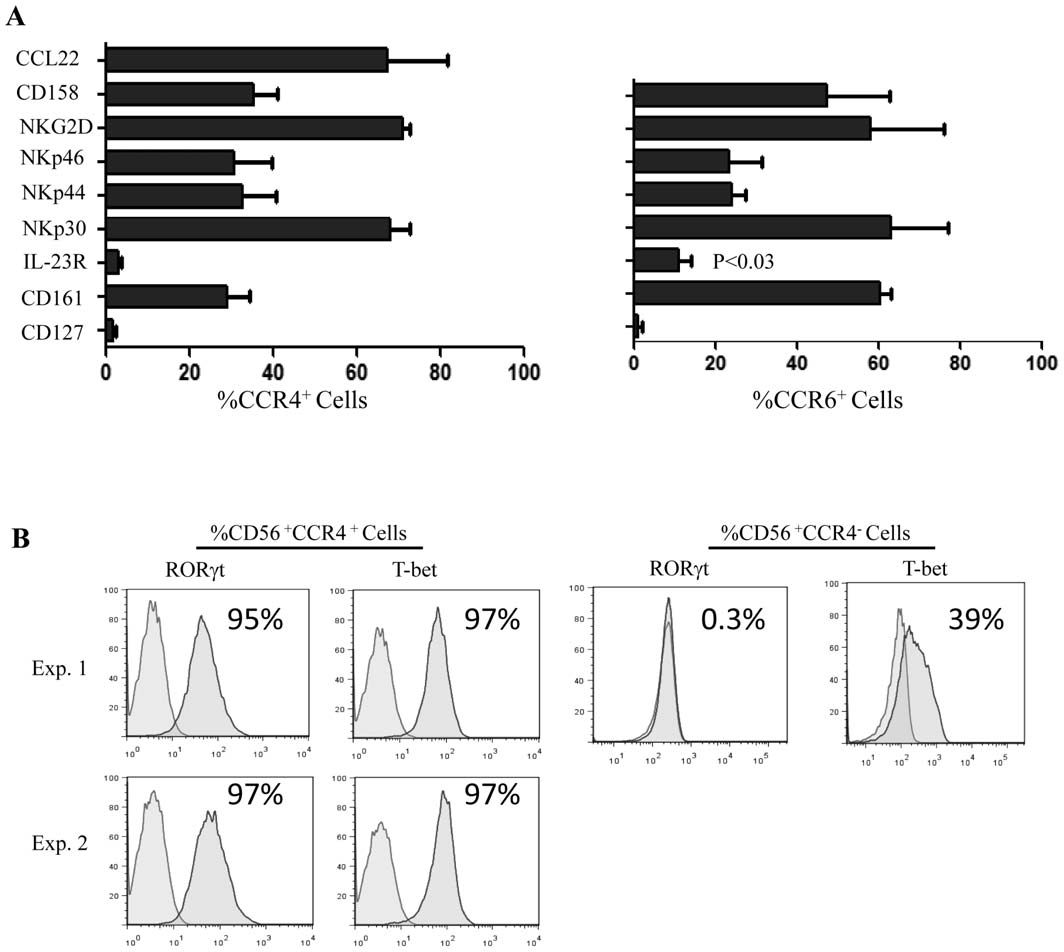
Expression of various markers by NK17/NK1 cells. A) IL-2-activated CD56^+^ NK cells were sorted by antibody-coated beads into CCR4^+^ or CCR6^+^ and then labeled with antibodies to various cell markers. The numbers are generated from 3 donors and are shown as mean±SEM. P value compares the frequency of CCR4^+^ and CCR6^+^ positive cells expressing IL-23R. B) CD56^+^ NK cells were sorted into CCR4^+^ or CCR4^−^and then labeled intracellularly with antibodies to RORγt and T-bet. Background using isotype control antibodies are shown in black. Numbers indicate the percentage of positive cells.

To gain insights into the expression of NK cell markers among different NK cell subsets based on their chemokine receptor possession, we double sorted IL-2-activated NK cells, first into CD56^+^ and then into CCR4^+^ or CCR6^+^ cells by antibody-conjugated beads. These subsets were labeled with antibodies to various surface receptors. The results show that both NK cell subsets expressed the mature NK cell molecules NKp30, NKp44, NKp46, NKG2D, CD158 and CD161, but lacked the expression of immature cell marker CD127. The most obvious difference between the two subsets is the expression of IL-23 on the surface of CCR6^+^ NK cells and not on NK17/NK1 (CD56^+^CCR4^+^) cells (P<0.03, Figure 4A). Notably, NK17/NK1 cells expressed the ligand for CCR4, i.e. CCL22/MDC, suggesting that this chemokine may play a role in the maintenance and/or survival of these cells.

To gain further insights into the molecular pathways involved in the production of IL-17 and IFN-γ, we examined the expression of the transcription factors RORγt and T-bet important for the secretion of IFN-γ and IL-17, respectively [22], [23]. Hence, sorted CD56^+^CCR4^+^ NK cells were labeled with anti-T-bet or anti-RORγt. Interestingly more than 94% of CD56^+^CCR4^+^ NK cells stained with antibodies to RORγt or T-bet (Figure 4B), suggesting that these transcription factors are important for the production of IL-17 and IFN-γ by these cells. On the other hand, CD56^+^CCR4^−^ cells did not express RORγt but about 40% of them expressed T-bet (Figure 4B).

### NK17/NK1 cells are increased in the cerebrospinal fluid (CSF) of multiple sclerosis (MS) patients

**Figure 5:**
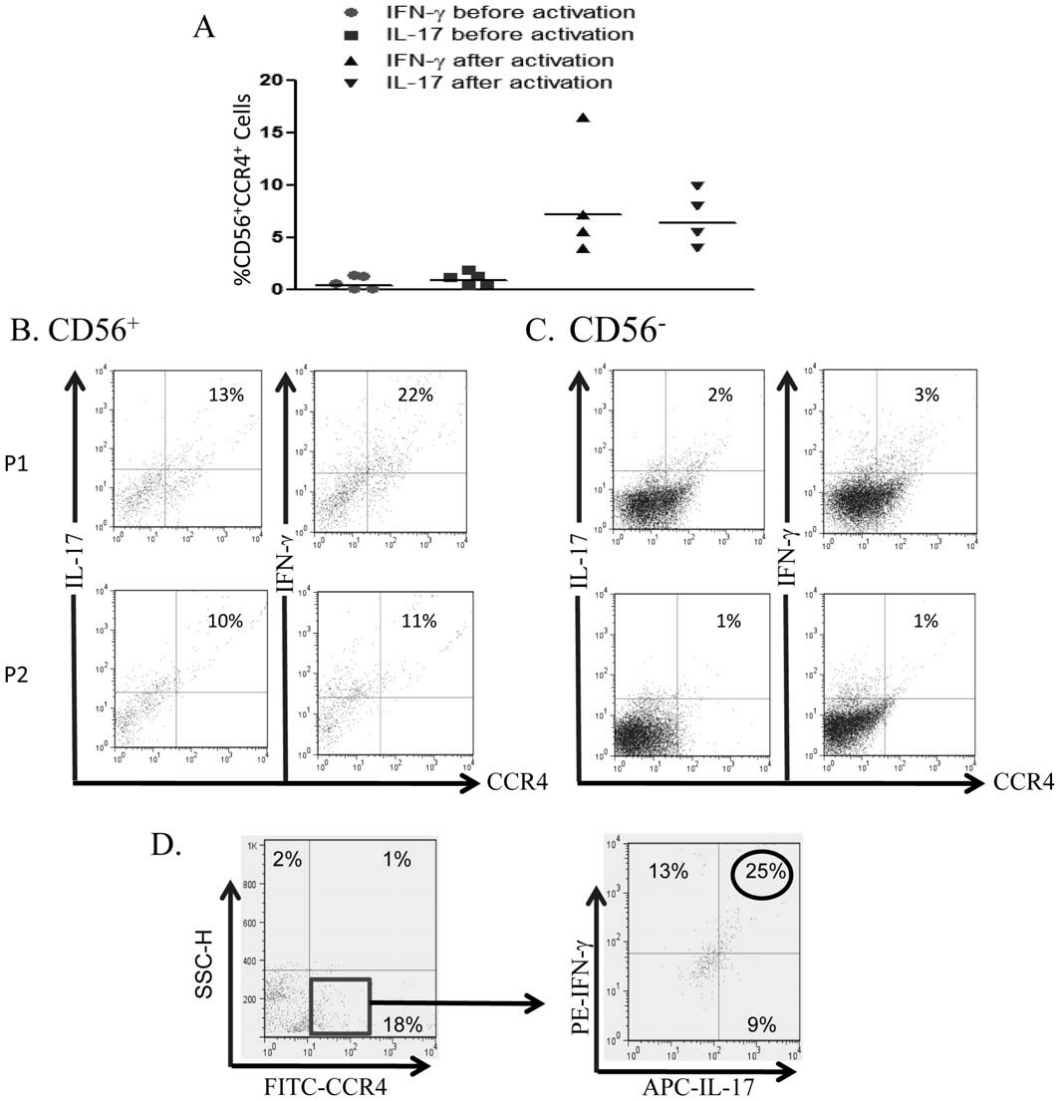
NK17/NK1 cells are abundant in the CSF of MS patients. A) NK cells were isolated from the blood of MS patients. CD56^+^ cells were isolated and labeled with FITC-conjugated anti-CCR4 and intracellularly with PE-conjugated anti-IL-17 and anti-IFN-γ. Each dot represents the percentage of CD56^+^CCR4^+^ cells producing IL-17 and IFN-γ from an individual patient before and after activation with IL-2. B) CSF from two MS patients (P1 and P2) were sorted into CD56^+^ NK cells and then labeled with FITC-conjugated anti-CCR4, PE-conjugated anti-IL-17, and PE-conjugated anti-IFN-γ. Numbers show percentages of cells producing IL-17 and IFN-γ. C) This is similar to panel B, except that CD56^-^ NK cells isolated from the same MS patients were examined. D) Cells from the CSF of a third MS patient were labeled with isotype control (not shown) or FITC-conjugated anti-CCR4 antibody and intracellularly with isotype control (not shown), or PE-conjugated anti-FITC and APC-conjugated anti-IL-17 antibodies. FITC-conjugated cells were gated (more than 99% positive for CCR4 expression), and examined for the production of intracellular cytokines. Numbers show the percentage of positive cells.

Both Th1 secreting IFN-γ and Th17 secreting IL-17 contribute to the pathogenesis of MS and EAE. Consequently, we examined the presence of NK17/NK1 cells in the blood of patients with MS. The results from five different patients show that NK17/NK1 cells were not spontaneously found in the blood, but were generated from the peripheral blood of four patients examined upon activation with IL-2, although their frequencies were less than in normal blood (Figure 5A). Of note, blood samples were collected from MS patients age 25–53 years old, which were not different from samples collected from healthy donors. We have also included CSF from MS patients due to the possibility that this may give us an idea about the role these cells might play in a diseased organ. After isolation of non-activated CD56^+^ cells from the CSF of two MS patients, CCR4^+^ cells secreting both IL-17 and IFN-γ were abundant (Figure 5B). The frequency of CD56^+^CCR4^+^ cells (NK17/NK1 cells) was about ten-fold higher than CD56^−^CCR4^+^ cells found in the CSF (Figure 5B vs. 5C), and more than twenty-fold the numbers of non-activated CD56^+^CCR4^+^ found in the peripheral blood of MS patients (Figure 5B vs. 5A). Also, we managed to obtain enough cells from the CSF of a third MS patient which were labeled with FITC-conjugated anti-CCR4 and intracellularly with PE-conjugated anti-IFN-γ and APC-conjugated anti-IL-17. The results in Figure 5D demonstrate that about 25% of CCR4^+^ NK cells produced both IL-17 and IFN-γ. It is highly plausible that NK17/NK1 cells may migrate from the periphery into the CSF aided by the CCL22/CCR4 axis, and are polarized in the brain due to inflamed local microenvironment that may contribute to their generation. CSF of normal individuals was not collected due to ethical considerations. However, NK cells are either not found or found in very low numbers in the brain of normal mice [24].

## Discussion

We describe here a novel subset of human NK cells phenotypically characterized as CD56^+^ CCR4^+^ RORγt^+^ T-bet^+^ IL-23R^−^, expressing mature NK cell markers, the ligand for CCR4, and producing IL-17 and IFN-γ. Earlier findings show that NK cells can be classified into different subsets based on their expression of chemokine receptors [4], but the functions of these subsets are not known. Our results are the first to show that a subset of human NK cells expressing a specific chemokine receptor perform distinguished function related to the production of inflammatory cytokines.

Because NK17/NK1 cells differ from both Th17 and NK22 since they do not express CCR6 or IL-23R, they may represent a distinct subset of NK cells. NK cells should have multiple specialized lineages or subsets since they perform multiple tasks [25]. Further, NK cells have memory similar to adaptive T cells indicating that various subsets or lineages of these cells may be recalled in response to various pathogens or cytokines. Under pathological conditions where IL-2 is released, we anticipate that NK17/NK1 cells predominate, which may affect the microenvironment through the release of IL-17 and IFN-γ. Intriguingly however, is the lack of any subset of NK cells examined in this study that can secrete only IFN-γ. This includes CD56^+^ or CD56^−^ cells that also express CCR6^+^, CCR7^+^, CCR9^+^, or CXCR4^+^. The only cells that secrete IFN-γ also secrete IL-17 (i.e. NK17/NK1 cells). It is either that cells of this subset are the only producers of this cytokine, or that IFN-γ is released by other NK cell subsets not examined in this study. The observation that CD56^+^ cells devoid of CCR4 express T-bet transcription factor suggests that cells lacking CCR4^+^ as well as CCR6^+^, CCR7^+^, CCR9^+^, or CXCR4^+^ might also secrete IFN-γ.

The finding that NK17/NK1 cells do not express IL-23R, whereas CCR6^+^ cells express it but do not produce IL-17 or IFN-γ, suggests that in the periphery the role of IL-23 may be replaced with available cytokines such as IL-2. Hence, IL-23 inducing the release of IL-17 may be a property of NK cells found at mucosal sites as a response to microbial infections, [5]–[7], whereas the rules of regulation are different in the periphery and at inflamed sites where more mature NK cells predominate. Plausibly, NK cells exposed in the periphery to IL-2 generate NK17/NK1 cells that express CCR4, whereas those that are exposed to IL-23 in the mucosa generate NK22 cells that express CCR6. Further, NK17/NK1 cells present in the periphery express RORγt^+^ and T-bet^+^ transcription factors, which facilitate their secretion of IL-17 and IFN-γ.

The role of NK cells in MS/EAE is controversial and it is not yet clear whether NK cells ameliorate or exacerbate the disease [10], [26]. We recently reported that administration of glatiramer acetate (GA) a drug used to treat MS patients, reduced EAE clinical score in SJL mice corroborated with isolating NK cells that expressed high killing potential against immature or mature dendritic cells [24]. In addition, administration of anti-Tac (Daclizumab) antibody to MS patients ameliorated the disease, associated with induced expansion and activation of CD56^+^ NK cells [27]. These findings demonstrate that NK cells may contribute to the therapeutic efficacy of these drugs.

In summary, we have identified a new subset of human NK cells that has not been previously recognized. The cells of this subset are designated as NK17/NK1 cells because they produce and secrete IL-17 and IFN-γ. They are generated from the peripheral blood of healthy individuals as well as MS patients upon activation with IL-2, and are abundant in the CSF of MS patients. The precise role that NK17/NK1 play in MS and other autoimmune diseases is currently under investigation.
